# Developing Soft Skills for Sustainable Community Pharmacy Practice Through a Competency-Based Modular Programme

**DOI:** 10.3390/pharmacy13040110

**Published:** 2025-08-20

**Authors:** Ivana Zimonjić, Lazar Dražeta, Valentina Marinković, Tatjana Milošević

**Affiliations:** 1 Department of Social Pharmacy and Pharmaceutical Legislation, Faculty of Pharmacy, University of Belgrade, 11221 Belgrade, Serbia; valentina.marinkovic@pharmacy.bg.ac.rs; 2 Galenika ad Beograd, 11080 Belgrade, Serbia; 3 Department of Management, Faculty of Business Economy, Singidunum University, 11000 Belgrade, Serbia; ldrazeta@singidunum.ac.rs; 4 Pharmacy Melem, 15000 Šabac, Serbia; tanja.melem@gmail.com; 5 EduMed, 15000 Šabac, Serbia

**Keywords:** pharmacy practice, continuing pharmacy education, competencies, training and development

## Abstract

This study explored a competency-based soft-skills programme supporting evolving community pharmacy professionals’ roles and sustainable practice in Serbia. Four researchers with academic and practice backgrounds developed the programme using healthcare guidelines and the International Pharmaceutical Federation’s competency framework. The process involved defining objectives, selecting methods, designing and organising activities, accreditation, and evaluating outcomes based on the Kirkpatrick model. From January 2021 to March 2025, the “Galenika Academy” was implemented through webinars, accredited tests, onsite courses, and a mobile application. Satisfaction was assessed via a validated online questionnaire among participants attending ≥80% of sessions, following evaluation of attendance and test performance. The programme reached 5107 participants, 10,427 webinar views, and 8252 test completions. The “Galiverse” mobile app, launched in February 2023, had 5558 users by March 2025. The most attended webinar was “Burnout” (787). Average test success was 82.9%, with 95.3% for “Resilience” and 61.0% for “Team Management.” Satisfaction was 95.5% for content, 94.2% for quality, 92.3% for materials, 77.1% for the application, and 96.3% would recommend it. Among those reporting improved resilience, 96.9% believed it could positively impact pharmacy operations. Pharmacists found the programme relevant and effective. Further research is needed to evaluate its impact on practice and patient outcomes.

## 1. Introduction

Since the COVID-19 pandemic, there has been increasing discussion about the healthcare workforce crisis [1,2]. Issues such as staff shortages, burnout, the second victim phenomenon, and employee turnover have become global concerns [3,4,5,6]. By aligning the needs of employees and the healthcare system, it is possible to enhance the capacities of both and support a sustainable healthcare system [7]. In 2020, the World Health Organisation (WHO) introduced the World Patient Safety Day Goals 2020–2021 to mark Patient Safety Day. Goal 2 focuses on reducing work-related stress and burnout, while Goal 4 aims to prevent violence against healthcare professionals [8]. In 2021, WHO launched the Global Patient Safety Action Plan 2021–2030, which, for the first time, recognised the importance of soft skills (non-technical, cognitive, and interpersonal [9]) in healthcare practice and patient safety [10]. Soft skills refer to a broad set of personal and interpersonal competencies—such as communication, teamwork, adaptability, leadership, and emotional intelligence—that are transferable across professional contexts and essential for effective collaboration and service delivery in healthcare [9]. These skills complement technical competencies and contribute significantly to safe, person-centred care [10]. Furthermore, in collaboration with the International Labour Organisation, the WHO released the 2022 guide Caring for Those Who Care [11], providing strategies for creating supportive environments for healthcare workers.

Although pharmacists’ roles as healthcare professionals are defined by clinical pharmacy and pharmaceutical care [12,13], they are often under-recognised by general practitioners [14]. Collaboration at the primary care level remains limited. A potential reason is the lack of funding mechanisms in many countries, where pharmacists are not employed within or reimbursed through national health insurance systems [15]. Despite these challenges, pharmacists’ roles in primary care are expanding, with advanced services like medication reviews and prescribing being implemented [16,17,18]. The WHO UNESCO FIP Pharmacy Education Taskforce highlighted the need for additional pharmacist training to prepare for future practice [19]. As studies show reluctance among pharmacists to accept responsibility for therapy outcomes [20], a shift in educational approaches is essential to meet these evolving demands.

Traditional systems for continuous professional development (CPD) for pharmacists have focused on technical skills, with soft skills only recently gaining attention [21]. The International Pharmaceutical Federation (FIP) Competency Framework defines clusters of these skills for both early practitioners and educators, highlighting the need for competency-based education [22,23,24]. As advanced practice standards align with competencies, their development directly impacts practice quality and safety [25,26]. Adverse pharmaceutical care outcomes often stem from compromised standards, further underscoring the need to strengthen pharmacists’ soft skills [27,28].

In Serbia, pharmacy technicians complete a four-year secondary school programme, while pharmacists graduate from an integrated Master’s degree programme. Both professional categories are required to pass a national licensing exam before entering practice. They are required to renew their professional licences every seven years by collecting a total of 140 continuing education points, with at least 10 points per working year [29]. Beyond initial qualifications, additional education contributes to broader knowledge and demonstrates a commitment to lifelong learning. Currently, around 15,000 pharmacy professionals actively involved in healthcare services are obliged to participate in CPD to retain their licences, making the content and accreditation of such programmes essential [30,31]. For competency assessment, the National Competency Framework is utilised. The initial version was a direct translation of the FIP framework [32]; however, since late 2024, a revised edition has been implemented, incorporating local context and providing a clear rating scale [33]. Competency indicators are rated descriptively from 1 to 4: always (level 4), often (level 3), occasionally (level 2), or never (level 1). Assessments may be conducted via self-evaluation, team-based evaluation, blinded assessment, or through specialised centres. Despite pharmacists self-assessing these skills as well-developed, CPD programmes in Serbia have traditionally excluded these topics and lacked accreditation for licence renewal [34]. This intervention aimed to support community pharmacy professionals in Serbia in evolving roles and pharmacy operations sustainability through a competency-based modular development programme.

## 2. Materials and Methods

### 2.1. Study Design and Tools

The research was designed as an interventional study with community pharmacy professionals at the national level in Serbia. Academic and practice experts developed the modular programme, named “Galenika Academy”, based on a literature review and practical experience, in line with the healthcare guideline *Intervention Mapping: A Process for Developing Theory and Evidence-Based Health Education Programs* [35] and the International Pharmaceutical Federation’s competency framework [25]. The process included the following: (i) defining programme objectives according to previous needs assessment, (ii) selecting theory-based intervention methods and practical strategies, (iii) designing and organising online lectures, tests, and courses, (iv) accreditation by national bodies, and (v) generating a plan for evaluating programme outcomes using the Kirkpatrick model [36].

The Ethics Committee of the Pharmaceutical Chamber of Serbia approved the evaluation of this programme (Reference number: 75/5-4, dated 8 October 2024) with the previous consent for data usage from the association for continuous medical education EduMed and Galenika a.d. Beograd (Reference number: 380, dated 22 March 2024).

**Figure 1:**
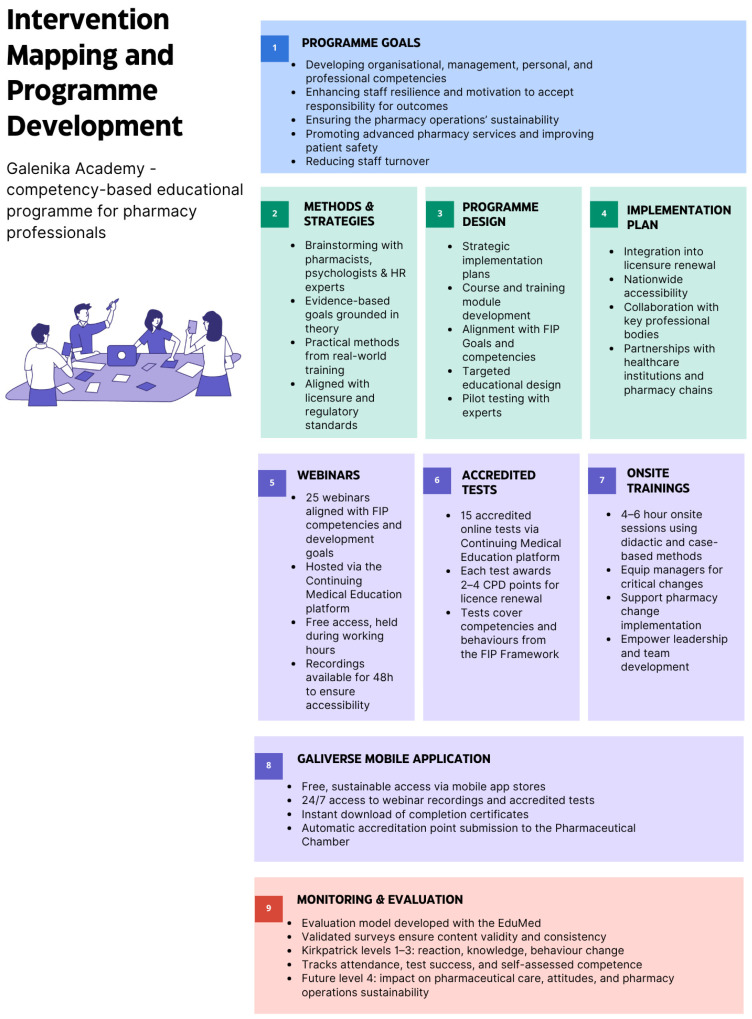
Development of a competency-based educational programme for pharmacy professionals.

The study followed the *Template for Intervention Description and Replication (TIDieR)* guide [37] and the *Defined Criteria to Report Innovations in Education (DoCTRINE) guidelines* [38]. The complete method is illustrated in Figure 1 for detailed visual review.

#### 2.1.1. Defining Programme Objectives According to the Previous Needs Assessment

A group of five experts from academia and practice, including two pharmacists, a psychologist, a human resource manager, and a soft-skills trainer, assessed needs based on the available literature [27,34], regulatory changes [39,40,41], and the necessity to support pharmacists for advanced practice [25]. The programme objectives include the following: (i) developing organisational, management, personal, and professional competencies for pharmacists and pharmacy technicians in community pharmacies; (ii) enhancing staff resilience and motivation to accept responsibility for outcomes; (iii) ensuring the pharmacy operations sustainability; (iv) promoting advanced pharmacy services and improving patient safety; and (v) reducing staff turnover.

#### 2.1.2. Selecting Theory-Based Intervention Methods and Practical Strategies

The approach involved building on previous research and successful practices in training and CPD [42]. It drew from theoretical literature to address a solid conceptual foundation, while empirical training methodologies were applied to guarantee practical relevance. The programme also considered accreditation for professional licence renewal, aligning with regulatory requirements and previous practices [21,42] as well as fostering motivation for learning, ensuring its effectiveness and sustainability within the professional framework.

#### 2.1.3. Designing and Organising Online Lectures, Tests, and Courses

The strategic plan was designed collaboratively by the same multidisciplinary team to ensure alignment with local pharmacy practice needs. Course curricula, learning materials, tests, and training modules were developed in alignment with the FIP Development Goals and Competency Framework [22,43,44]. Educational activities were carefully designed to address competency clusters, specific competencies, and expected behaviours outlined in the FIP framework. These materials were pilot-tested with a group of five expert practitioners to improve their relevance and effectiveness before broader implementation.

#### 2.1.4. Specifying Adoption and Implementation Plans

The programme was accredited by the Health Council of Serbia and the Pharmaceutical Chamber and integrated into the professional licensure renewal system for nationwide accessibility. Collaboration was established with the Association for Continuous Medical Education (EduMed) and the Pharmaceutical Chamber of Serbia, and partnerships with healthcare institutions and pharmacy chains were formed to enhance credibility and participation. Most educational sessions were planned via EduMed’s online platform, aligning with previous research to maximise accessibility and efficiency [45], while on-site courses were tailored for smaller groups of pharmacy owners and key managers [46]. Industry support played a crucial role in programme implementation. To support the sustainability of the programme and broader education availability, a mobile learning application “Galiverse” was developed, offering pharmacists 24/7 access to video recordings of previous educational sessions and accredited licence renewal tests (free for pharmacists and available in mobile application stores) [47].

#### 2.1.5. Generating a Plan for Evaluating Programme Outcomes

A programme evaluation plan was developed using the Kirkpatrick model [36] at three levels, assessing participants’ satisfaction and engagement, knowledge, skills, and behaviour change. Engagement was assessed through analysis of participation data from the EduMed platform, the official programme organiser, while knowledge was evaluated using accredited tests approved by the Pharmaceutical Chamber of Serbia for professional licence renewal, in line with national continuing professional education regulations. Regarding knowledge assessment, the online tests were primarily in the form of multiple-choice questions (MCQs), with occasional case-based and scenario-based questions to support reflection and application of knowledge.

A validated self-reported satisfaction questionnaire was developed by a group of five experts (two in pharmaceutical practice, one in psychology, and two in human resources management). Five practice experts validated this questionnaire according to Polit and Beck’s guideline [48], with the final version consisting of 14 questions on the created programme and 7 demographic questions (Supplementary Materials S1). Satisfaction-related questions were measured using a 5-point Likert scale (1—Very dissatisfied, 2—Dissatisfied, 3—Neutral, 4—Satisfied, 5—Very satisfied). Due to the nature and scale of the programme, on-the-job assessments were not feasible. To address this, assessment of competencies and behavioural change was conducted through self-assessment, following the National Competency Framework for Pharmacists, which recognises self-assessment as a valid method [32,33]. A satisfaction questionnaire was distributed to assess participants’ satisfaction, attitudes, and self-reported competence improvement.

### 2.2. Setting, Sampling, and Recruitment

Recruitment was purposive using snowball sampling [49] with email invitations (that enabled participants to forward the registration link to colleagues) to all members from the EduMed database (15,000 pharmacy professionals), ensuring diversity in sex, age, educational level, working position, and geographic location. Recruitment began in January 2021 and was repeated for each educational module until March 2025. Participants were required to have the following: (i) an active working licence issued by the Pharmaceutical Chamber of Serbia, and (ii) a working position in a community pharmacy at the moment of attending the programme.

### 2.3. Data Collection

Data collection was conducted by two researchers (I.Z. and T.M.), analysing the EduMed database. At the start of the registration on the platform, the pharmacy professionals agreed to the membership conditions. They consented to the use of their personal data to participate in the research, previously anonymised and coded. The previously validated satisfaction survey was sent online via the EduMed platform to the email addresses of participants who attended 80% or more of the available educational sessions. Participants were eligible for the survey if they had completed more than 80% of all offered webinars (representing approximately 80% of the total programme content and duration) and more than 80% of all accredited tests (approximately 15% of the programme). On-site courses, accounting for about 5% of the programme, were optional and not part of the inclusion criteria. This approach ensured that respondents had substantial engagement with both the instructional and assessment components of the programme. The overall monitoring and data collection period spans from January 2021 to March 2025.

#### 2.3.1. Online Education: Webinars Accessible to the Entire Pharmacy Community

Between January 2021 and December 2023, 25 online webinars were delivered via the EduMed platform, following the FIP competency framework and covering clusters such as Organisation and Management and Professional/Personal, with additional impact on Pharmaceutical Public Health and Emergency Response (for details, see Supplementary Materials S2). The sessions addressed topics like resilience, burnout, second victims, communication, teamwork, performance management, employee development, risk management, business decision-making, professionalism, and ethics. Participants were invited to webinars using the EduMed database, which covers the entire pharmacy community in Serbia.

#### 2.3.2. Accredited Tests: Available to All Pharmacy Professionals

In the period from January 2021 to March 2025, 15 online tests were accredited by the Health Council of Serbia for pharmacist licence renewal. For the first time in Serbia, training on soft skills for pharmacy professionals was accredited. The tests covered specific competencies from the FIP framework, most aligned with the webinar topics. Key themes included pharmacy as a business system, business skills requirements, time management, teamwork, performance management, decision-making in pharmacy practice, negotiation, practice research, and employee development. Tests were available for a time of two or three years, depending on the topic. Each test contained 20–40 questions, required 2–4 h of study from pre-provided materials, and took 30 min to 1 h to complete (for details see Supplementary Materials S3). Participants who scored ≥60% on the final knowledge assessment passed the test. Tests awarded 2-4 CPD points for licence renewal. Access codes for the tests were sent to participants after each webinar, and from 2023, following the launch of the “Galiverse” learning mobile application, the tests became available to all pharmacists via the application throughout the entire accreditation period.

#### 2.3.3. Onsite Training for Pharmacy Managers

Between March 2021 and December 2024, five onsite training sessions were organised for pharmacy owners and managers of key pharmacy chains using the didactic and case-based methods. Invitations for the onsite courses were primarily extended to pharmacy managers who had previously completed more than 80% of the webinars delivered up to that point, ensuring that participants in these sessions had substantial prior engagement with the programme’s core content. The aim was to empower and develop participants responsible for on-site staff development, thereby influencing the pharmacy operations sustainability. Topics included building effective relationships, leadership, performance management, employee development, change management, and time management. The educational objectives and expected outcomes for each training session are summarised in Table 1. The sessions were designed for smaller participant groups, following the training methodology and available resources.

The webinars, tests, and onsite courses were not interdependent. They were independently designed and offered, with thematic overlaps but without prerequisite requirements. Managers with over 80% webinar attendance were invited to onsite trainings to prioritise the most engaged participants, given limited resources. This flexibility allowed participants to engage with the content that best suited their current professional needs. As the programme ran over four years and included a large volume of material, full participation was neither expected nor required. The structure was designed to allow flexibility, enabling pharmacists to choose topics and formats that best aligned with their individual needs and availability.

### 2.4. Data Analysis, Interpretation, and Storage

All raw data from the EduMed platform was coded and stored in Microsoft Word and Excel. The lead researcher ensured secure electronic storage with restricted access to personal computers. Descriptive statistics were conducted using SPSS software (SPSS v29.0 for Windows, SPSS Inc., Chicago, IL, USA). Data analysis included attendance, test performance, and exam success rates. Satisfaction survey data of participants attending ≥80% of sessions were analysed separately. Statistical significance was set at *p* < 0.05. The chi-square test examined the relationship between programme attendance and pharmacy professionals’ outcomes with Phi, Cramer’s V, and the contingency coefficient to assess the strength of relationships. To determine whether there were statistically significant differences between pharmacists and pharmacy technicians regarding their evaluation of the educational programme, a series of crosstabulations was conducted. Comparisons were made for demographic variables, perceived improvement of competences, and responses at all three levels of evaluation (reaction, learning, behaviour). Phi and Cramer’s V were used to assess the strength and significance of associations.

## 3. Results

### 3.1. Demographic Characteristics of the Programme Participants

Of the total participants, 5107 were unique individuals, representing approximately 34% of the entire pharmacy community.

In this sample (*n* = 5107), most participants were female (91.8%) and pharmacists (67.2%), employed in private pharmacies (84.5%), mostly in large cities (45.2%). The response rate for invitation calls was 34.1% (from an EduMed base of 15,000 participants: 93.5% female, 60.9% pharmacists, 85.7% employed in private pharmacies, 46.8% from large cities), with 5107 responding and engaging with the programme. Of the 5107 programme participants, 869 completed over 80% of the webinars, 766 completed over 80% of the accredited tests, and 20 completed over 80% of the onsite courses. In total, 61 participants attended at least one onsite course, with attendance ranging from all five courses (*n* = 20) to only one course (*n* = 3). Around 15% of these participants (*n* = 766) completed more than 80% of both the offered webinars and the accredited tests, representing those who attended over 80% of all educational sessions. Detailed participation summary for all modules by completion rate and access platform is provided in Table 2.

### 3.2. Demographic Characteristics of the Satisfaction Survey (Participants Who Attended ≥80% of the Education)

The satisfaction survey received a 29% response rate (*n* = 222). Sociodemographic data for 222 respondents show an average age of 43 years and 17 years of work experience. The majority were female (91.4%), with 48.2% holding a master’s degree. Most participants were pharmacists in community pharmacies (66.2%) and worked in private pharmacies (87.0%). Detailed sociodemographic information is provided in Table 3.

### 3.3. Participant Engagement and Performance Outcomes

#### 3.3.1. Online Education: Webinars Accessible to the Entire Pharmacy Community

During the whole webinar live programme period (January 2021–December 2023), a total of 5107 participants reached 10,427 participations in the webinars, with the most popular being “Small school of resilience for healthcare professionals—burnout” (787 participants), “Why is time management important for pharmacists in pharmacies?” (752 participants), and “Risk management in pharmaceutical care” (734 participants). Other well-attended sessions included “Why digital communication and leadership are important for pharmacists?” (650 participants) and “Small school of resilience for healthcare professionals—second victims” (692 participants). All topics are summarised in Table 4, with a mapping of competence clusters and specific competencies available in Supplementary Materials S2.

#### 3.3.2. Accredited Tests: Available to All Pharmacy Professionals

Additionally, for the period from January 2021 to March 2025, 5107 participants accessed 8252 tests. As shown in Table 2, Table 3 and Table 4, the evaluation of the accredited education programme reflects strong participant engagement. Notably, 928 individuals participated in the “Teamwork of health professionals as a prerequisite for patient safety” test, with 722 achieving a pass rate of 77.8%. The “Resilience of health professionals as a prerequisite for patient safety” test had the highest success rate, with 482 out of 506 passing (95.3%). On the other hand, the “Creating and managing teams in pharmaceutical organisations” test saw a success rate of 61.0%, with only 292 out of 479 participants passing.

All tests are summarised in Table 5, with detailed information on each test available in Supplementary Materials S3.

#### 3.3.3. Onsite Trainings for Pharmacy Managers

In the total period from March 2021 to December 2024, 61 unique participants attended the management onsite didactic and case-based training courses, with 20 managers completing all five modules. The most attended training sessions were Effective Leadership and Change Management, each with 30 participants. These were followed by Building Effective Relationships, Performance Management and Employee Development (each with 28 participants), and Change Implementation (with 25 participants). The manager group included 61 pharmacists: 48 female (78.7%) and 13 male (21.3%), with a mean age of 46 years (range: 29–60, SD = 8.5) and an average of 20 years of work experience (range: 1–35, SD = 9.5). Twelve (19.7%) were involved in healthcare service provision, in addition to a managerial role. Regarding managerial level, 30 (49.2%) were low-level and 19 (31.1%) high-level managers. Fifty-six (91.8%) were from private pharmacy chains, while 5 (8.2%) worked in state-owned pharmacies. All participants (*n* = 61) watched more than 80% of the webinars. Twenty-seven (44.3%) participants completed more than 80% of the knowledge tests with a mean success rate of 89.8%. According to the post-test questionnaire, 91.8% of participants agreed that the programme improved their competencies, and 96.4% stated they would recommend it to colleagues aiming to develop leadership within their teams.

### 3.4. Galenika Academy Satisfaction Questionnaire Results

Among the 222 respondents, overall satisfaction with the Galenika Academy programme was rated very high, with a mean score of 4.8 (SD = ±0.59; range: 1–5). Participants expressed equally high satisfaction with the quality and selection of lecturers (M = 4.8, SD = ±0.61), while the preparatory materials for accredited tests were rated slightly lower but still favourably (M = 4.6, SD = ±0.80). Satisfaction with the functionality and usability of the mobile application received a lower score (M = 4.2, SD = ±1.20), though it remained within a positive range. An overwhelming majority of participants (*n* = 214; 96.3%) reported that they would recommend the programme to their colleagues. In addition, most respondents completed the accredited tests, further confirming the practical value and applicability of the educational content. In terms of self-assessed outcomes, 213 participants (95.9%) agreed that the programme had improved their competencies. Changes in behaviour related to resilience were rated positively, with a mean score of 4.3 (SD = ±0.91). Satisfaction with the programme’s impact on pharmacy operations sustainability was similarly high, with a mean score of 4.5 (SD = ±0.77). Detailed survey results in relation to the Kirkpatrick model of evaluation are provided in Table 6.

Chi-square results indicated a significant relationship between participation and participants’ outcomes (*p* < 0.001). Of those who were aware that they attended 80% (*n* = 194) of the training, 84% (*n* = 163) were very satisfied with the content, 85.1% (*n* = 165) with the quality and choice of speakers, and 67.3% (n = 115) of those aware of the mobile application (*n* = 171) were very satisfied with it. Among those who were very satisfied with their competency improvement (*n* = 129), 92.2% (*n* = 119) were highly satisfied with the programme’s content, 90.7% (*n* = 117) with the test preparation materials, 79.8% (*n* = 103) with the mobile application, and 96.9% (*n* = 125) with the programme’s impact on business sustainability. Detailed statistical results are presented in Supplementary Materials S4.

Crosstabulation analyses between pharmacists and pharmacy technicians revealed no statistically significant differences in demographic characteristics or responses across the three levels of evaluation (reaction, learning, behaviour). An exception was observed in the perception of competence improvement, where a significant association was found between job position and perceived competence enhancement (Cramer’s V = 0.139, *p* = 0.038). While 100% (*n* = 75) of pharmacy technicians reported perceived improvement, 94.1% (*n* = 138) of pharmacists responded positively. However, the strength of the association was weak. For all other variables, including satisfaction with content and format, knowledge gained, and application of new skills in practice, no significant differences were identified between the two groups (all *p* > 0.05). Among participants in onsite training who attended more than 80% of both webinars and accredited tests, the mean test success rate was 89.8%. When compared with the overall average success rate of 82.9% for all test-takers and 84.7% for those who attended over 80% of webinars and tests, this difference was not statistically significant (Phi = 1.414, Cramer’s V = 1.000, Contingency Coefficient = 0.816; all *p* = 0.199).

## 4. Discussion

The “Galenika Academy” programme has effectively addressed a wide range of competencies within the clusters of organisation and management and personal and professional competencies, with elements of public health from the FIP framework. Over its four-year existence, more than a third of the pharmacy workforce in Serbia has engaged with the offered training, acquiring knowledge, initiating skills, and earning CPD points for licence renewal. The demographic characteristics of the programme participants closely mirror those of the recruitment database, which itself reflects the broader pharmacist population in the country [30,31]. As the database includes licensed pharmacists from diverse regions and practice settings, the sample can be considered representative in terms of key demographic variables such as sex, age, and type of pharmacy. The proportion of participants who attended over 80% of the sessions is considered satisfactory and exceeds expectations, especially given the programme’s complexity, duration, and non-mandatory nature.

According to Kirkpatrick’s model of training evaluation, participants expressed a very high level of satisfaction with the programme (Level 1—Reaction), particularly regarding the content, quality, and selection of lecturers. Satisfaction with the mobile application, while still high, was somewhat lower, which may be attributed to factors such as participants’ digital literacy, age, familiarity with mobile-based learning formats, and the technical features and user experience of the application itself. Nevertheless, the overall reception of the application remained positive. The majority of respondents indicated that they would recommend the programme to colleagues interested in developing similar skill sets. In terms of learning outcomes (Level 2—Learning), a notably high percentage of participants passed the knowledge assessments they undertook and reported perceived improvements in their competencies. Furthermore, satisfaction with behavioural change (Level 3—Behaviour) and the application of newly acquired knowledge and skills in practice was exceptionally high, suggesting strong transfer of learning into professional settings. Cross-tabulation analysis highlights the impact of the programme on satisfaction and self-perceived competence improvement, revealing clear links between programme participation and its positive effects on pharmacists and pharmacy practice.

The highest proportion of participants attended the webinars, which may be attributed to their accessibility—both in real time during scheduled sessions and on-demand for 48 h afterwards, and later via the mobile application. While participation in knowledge tests was also high, it was slightly lower than for webinars. This may be due to the requirement that tests be passed following access to the learning material, with results submitted directly to the professional development records within the Pharmaceutical Chamber or the Chamber of Health Technicians of Serbia. Nonetheless, the accreditation of these activities and the opportunity to collect CPD points for licence renewal had a positive impact on test participation. On-site training courses for managers generated significant interest; however, the number of participants was limited due to budgetary constraints. Analysis of test performance indicated that those who attended over 80% of the educational activities (webinars and accredited tests with preparation materials) and completed the satisfaction survey showed higher success rates in knowledge tests than those who only attended preparation materials for exams. Although each activity was designed as a stand-alone module, the overlap of key topics across multiple learning formats appeared to enhance knowledge retention and skills development.

The most frequently attended webinars addressed topics such as burnout, time management, risk management in pharmacy operations, digital communication, and secondary victimisation in pharmacy practice. These also aligned with the most attended knowledge tests, which focused on teamwork, communication, risk, and second victim support—reflecting some of the pressing challenges currently faced in pharmacy practice [27,50]. Manager participants demonstrated the greatest interest in leadership, performance and change management, and employee development, which is consistent with their strategic roles within pharmacy organisations.

The findings indicate a high level of consistency in the evaluation of the educational programme between pharmacists and pharmacy technicians. The only significant difference, though weak in strength, was in the self-reported improvement of competences, with all technicians reporting positive change. This may reflect different expectations or baseline confidence levels between the two professional groups. The overall lack of statistically significant differences suggests that the programme was perceived as equally relevant and practical across job roles. These results support the inclusive design of the training, which appears to meet the educational needs of both pharmacists and technicians alike.

The programme has aligned both its content and delivery methods with the latest trends, recommending changes in CPD activities for pharmacists worldwide [51,52]. Additionally, the incorporation of competencies relevant to emergencies reflects contemporary research trends [43,53]. Soft skills are increasingly recognised as essential for advancing pharmaceutical practice, as well as the impact of management and leadership competencies on employee satisfaction within pharmacies [54]. This, in turn, enhances work motivation and potentially improves outcomes in pharmaceutical care [50]. Global initiatives in healthcare professional development have also sparked research into the development of such skills, with several pilot projects already underway [55,56].

These results provide valuable guidelines for potential training and educational programmes for pharmacy professionals to be implemented for handling and preventing primary healthcare crises in the future. They could have a positive effect on employee satisfaction and the quality and sustainability of pharmaceutical practice. In Serbia, the programme has initiated the development of new undergraduate pharmacy courses, such as Professional Development, and supported research on how such education impacts both patient and pharmacist safety, as well as its role in shaping changes in undergraduate and continuing education [57]. It has also inspired internal development programmes tailored to specific pharmacy systems, which are currently being evaluated across all four Kirkpatrick levels in real-world practice settings.

Considering that community pharmacists are not yet included in educational-change research studies and development projects like other healthcare professionals, further recommendations for all stakeholders involved in education, regulation, and pharmaceutical development would be to engage with global research initiatives and implement and evaluate similar projects on an international scale and assess the impact on real-life practice.

### Strengths and Limitations

This study’s strengths include its rigorous methodology and, to the best of our knowledge, the first programme of its kind for pharmacy professionals. The intervention aligned with the FIP Competency Framework, improved digital adaptability, and offered a scalable, accredited model. A limitation of this study is the small satisfaction survey sample, excluding data from participants who attended less than 80% of the programme. Due to data protection, demographic data for the entire EduMed database was unavailable. On-the-job assessments were unfeasible, so self-assessment per the National Competency Framework was used. Future studies would benefit from a pre-/post-test design.

## 5. Conclusions

To our knowledge, this modular programme represents the first accredited initiative aimed at systematically developing soft-skills competencies among community pharmacy professionals. Participants’ self-assessments suggest improvements in resilience and pharmacy operations, with perceived benefits for patient care. Beyond individual outcomes, the programme has informed institutional changes in both undergraduate and continuing pharmacy education and has served as a model for internal development schemes now evaluated across all four Kirkpatrick levels in practice settings. These findings underscore the need for further longitudinal and practice-based research to assess the sustained impact on professional and patient safety.

## Figures and Tables

**Table 1:** Educational goals with expected outcomes of onsite training using didactic and case-based methods for pharmacy managers.

Training Topic	Educational Goals	Expected Outcomes
1. “Building effective relationships in pharmaceutical practice”	Enhance communication skills for effective collaboration. Understand how to build trust with colleagues and patients. Develop strategies for managing professional relationships. Learn how to improve teamwork and conflict resolution skills. Learn how to strengthen pharmaceutical care effectiveness in pharmacy practice.	Improved communication skills for effective collaboration. Building trust with colleagues and patients. Effective management of professional relationships. Improved teamwork and conflict resolution. Strengthen pharmaceutical care effectiveness in pharmacy practice
2. “Effective leadership in pharmaceutical practice”	Understand core principles of leadership in pharmacy practice. Develop skills to inspire and motivate team members. Enhance decision-making and problem-solving abilities. Foster a collaborative and productive work environment. Build strategies for effective team management and performance improvement.	Enhanced understanding of leadership principles in pharmaceutical practice. Improved ability to inspire and motivate pharmacy teams. Strengthened decision-making and problem-solving skills. Creation of a more collaborative and productive workplace. Effective implementation of team management strategies for improved performance.
3. “Performance management and employee development in pharmacy practice”	Understand the key concepts of performance management in pharmacy practice. Develop skills to assess and improve employee performance. Learn strategies for setting clear goals and expectations. Enhance abilities in providing constructive feedback and coaching. Foster a culture of continuous professional development and growth.	Improved understanding of performance management practices in pharmacy. Enhanced ability to assess and support employee performance effectively. Clear goal setting and expectation management for pharmacy teams. Improved skills in providing constructive feedback and coaching. Stronger culture of continuous professional development and growth within the pharmacy.
4. “Change management in pharmacy business”	Understand the principles and processes of change management in the pharmacy business. Develop skills to assess and manage change effectively within the pharmacy environment. Learn strategies for overcoming resistance to change and fostering acceptance. Enhance the ability to lead and support teams through change. Explore methods for evaluating the success of change initiatives in pharmacy business operations.	Enhanced understanding of change management principles and processes in the pharmacy business. Improved ability to manage and implement change effectively in the pharmacy setting. Increased competency in addressing resistance to change and promoting acceptance. Stronger leadership skills in guiding teams through change initiatives. Greater capability in evaluating and measuring the success of change efforts within the pharmacy business.
5. “Change implementation: the impact of (i) resilience, (ii) effective time management, and (iii) change management in the education system on success”	Understand the role of resilience in change implementation. Develop effective time management skills for managing change. Learn key change management strategies for successful implementation. Explore the impact of resilience and time management on educational success. Strengthen skills to adapt and lead in changing educational environments.	Improved resilience in managing and adapting to change. Enhanced time management skills for more effective change implementation. Better understanding and application of change management strategies. Greater ability to assess the impact of resilience and time management on success. Increased capacity to lead and succeed in dynamic educational settings.

Pharmacy managers included owners of small and medium-sized pharmacies as well as regional managers in large pharmacy chains (covering the entire country’s territory).

**Table 2:** Training participation summary by completion rate and access platform (*n* = 5107).

Completion Range (%)	Webinars (*n*) (%)	Web (*n*)	Galiverse (*n*)	Accredited Tests (n) (%)	Web (*n*)	Galiverse (*n*)	Onsite Courses (*n*) (%)
0–20%	111 (2.2)	89	22	450 (8.8)	90	360	3 (4.9)
20–40%	889 (17.4)	711	178	549 (10.7)	104	445	6 (9.8)
40–60%	1998 (39.1)	1594	404	2184 (42.8)	394	1790	14 (23.0)
60–80%	1240 (24.3)	868	372	1158 (22.7)	243	915	18 (29.5)
80–100%	869 (17.0)	773	96	766 (15.0)	115	651	20 (32.8)
Total	5107 (100)	4340	767	5107 (100)	1124	3983	61 (100)

Completion ≥ 80%—fully or mostly completed modules (videos watched, materials accessed, onsite training attended). Platform—primary access point for the module (system-tracked via login). Web—EduMed platform, Galiverse—mobile application platform. Number of participants (*n*) refers to the number of individuals falling into each completion category (numerator). The percentage of total participants (%) is calculated using the total number of individuals who started any training module (denominator). Numerator = number of participants in the given completion category. Denominator = total number of participants who began any training module.

**Table 3:** Demographic characteristics of the satisfaction survey (participants who attended ≥80% of the education) (*n* = 222).

Sociodemographic Variables	Categories	*n* (%)	Mean (Min–Max) (SD)
Age			43 (20–74) (11.6)
Years of work experience			17 (1–65) (11.9)
Sex	Female	203 (91.4)	
	Male	19 (8.6)	
Level of education	Secondary School	58 (26.1)	
	College—Bachelor’s Degree	14 (6.3)	
	University—Master’s Degree	107 (48.2)	
	Specialised Academic Studies	29 (13.1)	
	Health Specialisation	3(1.3)	
	Master’s or Doctorate	11 (4.9)	
Job position	Pharmacy technician in a community pharmacy	75 (33.8)	
	Pharmacist in a community pharmacy	147 (66.2)	
Type of institution	Private Pharmacy	193 (87.0)	
	State Pharmacy	29 (13.0)	
Size of the city	Rural area	22 (9.9)	
	Smaller town (up to 100,000 inhabitants)	90 (40.5)	
	Medium-sized city (100–200,000 inhabitants)	30 (13.5)	
	Large city (over 200,000 inhabitants)	80 (36.0)	

Eligible survey participants completed ≥80% of webinars (~80% of the programme) and ≥80% of accredited tests (~15%). Pharmacy technicians completed a 4-year secondary school, pharmacists an integrated Master’s; both passed a licensing exam. Additional education indicates broader knowledge and lifelong learning.

**Table 4:** Summary of webinars and participants in live sessions online (*n* = 5107).

Competence Cluster ^1^	Number of Topics	Webinar Topics	Number of Participants
**Professional/Personal**	11	”Small school of resilience for healthcare professionals—burnout”	787
		2. “Small school of resilience for healthcare professionals—second victims”	692
		3. “Small school of resilience for healthcare professionals—resilience”	452
		4. “Communication in healthcare”	438
		5. “Quality in pharmaceutical care”	433
		6. “Why are digital communication and leadership important for pharmacists?”	650
		7. “Pharmacy marketing mix and brand management”	171
		8. “Why is communication important for pharmacists in pharmacies?”	375
		9. “Why conflict management is important for pharmacists”	410
		10. “Why are professionalism and ethics important for pharmacy professionals?”	152
		11. “Why is research in pharmaceutical practice important for pharmacists?”	250
**Organisation and Management**	14	12. “Teamwork in healthcare”	563
		13. “Why is time management important for pharmacists in pharmacies?”	752
		14. “Risk management in pharmaceutical care”	734
		15. “Why is negotiation important for pharmacists?”	570
		16. “Why team development is important for pharmacists”	558
		17. “How to motivate employees in pharmacies?”	514
		18. “Why is a business plan important for a new service in a pharmacy?”	446
		19. “Why is business continuity management important in pharmacies?”	286
		20. “Pharmacy as a business system and the development of personal skills”	269
		21. “Tools for problem solving and business decision-making in pharmacies”	239
		22. “Why is performance management important for pharmacy professionals?”	189
		23. “Regulations in advertising in pharmacies”	167
		24. “Why is employee development important for the business of pharmacies?”	172
		25. “Project management in pharmaceutical practice”	158
**Total**			10,427

^1^ Individual competencies are categorised into clusters in alignment with the International Pharmaceutical Federation (FIP) Global Competency Framework—Early Career Training Version 2.

**Table 5:** Summary of accredited tests: topics, covered competencies, participant numbers, and pass rates.

Education-Accredited Test	Competence ^2^	Period of Availability	Number of Participants	Success Rate
“Pharmacy as a business system and development of personal abilities”	3.2 Human resources management	2 years	725	83.4%
2. “Time management in pharmaceutical organisations—pharmacies”	3.6 Workplace management	3 years	762	85.8%
3. “Creation and management of teams in pharmaceutical organisations—pharmacies”	3.2 Human resources management	3 years	479	61.0%
4. “Management of work performance in the pharmacy”	3.2 Human resources management	2 years	572	87.1%
5. “Teamwork of health professionals as a prerequisite for patient safety”	3.2 Human resources management	2 years	928	77.8%
6. “Decision-making and problem-solving in pharmaceutical practice”	3.3 Improvement of service	2 years	521	83.7%
7. “Business negotiation in pharmaceutical practice”	3.1 Budget and reimbursement	3 years	291	89.7%
8. “Risk management in pharmaceutical practice”	3.3 Improvement of service	2 years	555	84.7%
9. “Communication of health professionals as a prerequisite for patient safety”	4.4 Interprofessional collaboration	2 years	813	85.4%
10. “Research in pharmaceutical practice”	4.8 Quality assurance and research in the workplace	2 years	371	80.1%
11. “Healthcare professionals as second victims of negative outcomes in health care”	4.5 Leadership and self-regulation	2 years	682	79.8%
12. “Development of employees in the pharmacy”	4.2 Continuing Professional Development (CPD)	2 years	361	80.9%
13. “Resilience of health professionals as a prerequisite for patient safety”	4.5 Leadership and self-regulation	2 years	506	95.3%
14. “Burnout syndrome in health practice”	4.5 Leadership and self-regulation	2 years	546	94.5%
15. “Standards for establishing quality and safety in pharmaceutical practice”	4.8 Quality assurance and research in the workplace	2 years	140	90.0%
Total (number of participants, success rate for all tests)			8252	82.9%

Health Council of the Republic of Serbia approval and Pharmaceutical Chamber of Serbia accreditation for working licence renewal. Professional licences are renewed every seven years, requiring the accumulation of 140 continuing education points over the period, with a minimum of 10 points earned per working year. ^2^ Individual competencies are categorised in alignment with the International Pharmaceutical Federation (FIP) Global Competency Framework—Early Career Training Version 2. Success Rate—% of participants who pass the test. Participants who scored ≥60% on the final knowledge assessment passed the test.

**Table 6:** Survey results in relation to the levels of the Kirkpatrick model of evaluation (*n* = 222).

Kirkpatrick Level of Evaluation	Sociodemographic Variables	Categories	*n* (%)	Mean (Min–Max) (SD)	Programme Component
Level 1—Reaction	Participant satisfaction with the training				
		^3^ How satisfied are you with the content of Galenika Academy?		4.8 (1–5) (±0.59)	Webinars/Onsite courses
		^3^ How much are you satisfied with the quality of education and the choice of lecturers?		4.8 (1–5) (±0.61)	Webinars/Onsite courses
		^3^ How satisfied are you with the content of the materials provided for test preparation?		4.6 (1–5) (±0.8)	Accredited tests
		^3^ How satisfied are you with the mobile application?		4.2 (1–5) (±1.2)	Galiverse application
	Would you recommend an educational programme to colleagues who wish to develop professionally?	Yes	214 (96.3%)		Cross-cutting (all)
		No	8 (3.7%)		All
Level 2—Learning	Changes in knowledge, attitudes, and skills				
	Test score	≥60% (pass)	188 (84.7%)		Accredited tests
		<60% (fail)	34 (15.3%)		Accredited tests
	Do you perceive that the programme(s) you attended improved your competence?	Yes	213 (95.9%)		All
		No	5 (2.3%)		All
Level 3—Changed behaviour	Application of learned content in practice				
		^3^ How satisfied are you with the way you have incorporated resilience-building strategies into your professional practice?		4.3 (1–5) (±0.91)	All
		^3^ How satisfied are you with the way your behaviour has contributed to the success and sustainability of your organisation?		4.5 (1–5) (±0.77)	All

^3^ The following questions utilised a Likert scale (1—Very dissatisfied, 2—Dissatisfied, 3—Neutral, 4—Satisfied, 5—Very satisfied). Categories correspond to specific components of the Galenika Academy programme and indicate whether they relate to webinars, accredited tests, or onsite courses.

## Data Availability

The raw data supporting the conclusions of this article will be made available by the authors on request.

## Supplementary Materials

The following supporting information can be downloaded at: https://www.mdpi.com/article/10.3390/pharmacy13040110/s1, Supplementary Materials S1–S4: Galenika Academy satisfaction questionnaire.

## Abbreviations

The following abbreviations are used in this manuscript:

WHO	World Health Organisation
CPD	Continuous Professional Development
FIP	International Pharmaceutical Federation

## References

[1] Banse E., Petit G., Cool G., Durbecq J., Hennequin I., Khazaal Y., de Timary P. (2022). Case study: Developing a strategy combining human and empirical interventions to support the resilience of healthcare workers exposed to a pandemic in an academic hospital. Front. Psychiatry.

[2] Förster C., Füreder N., Hertelendy A. (2023). Why time matters when it comes to resilience: How the duration of crisis affects resilience of healthcare and public health leaders. Public Health.

[3] Busch I.M., Moretti F., Purgato M., Barbui C., Wu A.W., Rimondini M. (2020). Dealing With Adverse Events: A Meta-analysis on Second Victims’ Coping Strategies. J. Patient Saf..

[4] Busch I.M., Moretti F., Campagna I., Benoni R., Tardivo S., Wu A.W., Rimondini M. (2021). Promoting the Psychological Well-Being of Healthcare Providers Facing the Burden of Adverse Events: A Systematic Review of Second Victim Support Resources. Int. J. Environ. Res. Public Health.

[5] Busch I.M., Moretti F., Purgato M., Barbui C., Wu A.W., Rimondini M. (2020). Psychological and Psychosomatic Symptoms of Second Victims of Adverse Events: A Systematic Review and Meta-Analysis. J. Patient Saf..

[6] Burlison J.D., Quillivan R.R., Scott S.D., Johnson S., Hoffman J.M. (2021). The Effects of the Second Victim Phenomenon on Work-Related Outcomes: Connecting Self-Reported Caregiver Distress to Turnover Intentions and Absenteeism. J. Patient Saf..

[7] Kuhlmann E., Falkenbach M., Brînzac M.G., Correia T., Panagioti M., Rechel B., Sagan A., Santric-Milicevic M., Ungureanu M.I., Wallenburg I. (2024). Tackling the primary healthcare workforce crisis: Time to talk about health systems and governance—A comparative assessment of nine countries in the WHO European region. Hum. Resour. Health.

[8] Donaldson L.J., Neelam D. (2020). World patient safety day: A call for action on health worker safety. J. Patient Saf. Risk Manag..

[9] Mahadevan A., Rivera R., Najhawan M., Saadat S., Strehlow M., Rao G.V.R., Youm J. (2024). Assessing the Efficacy of a Novel Massive Open Online Soft Skills Course for South Asian Healthcare Professionals. J. Med. Syst..

[10] Astier-Peña M.P., Martínez-Bianchi V., Torijano-Casalengua M.L., Ares-Blanco S., Bueno-Ortiz J.M., Férnandez-García M. (2021). The Global Patient Safety Action Plan 2021–2030: Identifying actions for safer primary health care. Atención Primaria.

[11] World Health Organization (2022). Caring for Those Who Care: Guide for the Development and Implementation of Occupational Health and Safety Programmes for Health Workers: Executive Summary. https://www.who.int/publications/i/item/9789240040779.

[12] Dreischulte T., van den Bemt B., Steurbaut S., European Society of Clinical Pharmacy (2022). European Society of Clinical Pharmacy definition of the term clinical pharmacy and its relationship to pharmaceutical care: A position paper. Int. J. Clin. Pharm..

[13] Allemann S.S., van Mil J.W., Botermann L., Berger K., Griese N., Hersberger K.E. (2014). Pharmaceutical care: The PCNE definition 2013. Int. J. Clin. Pharm..

[14] Chong J.B.K., Yap C.Y.H., Tan S.L.L., Thong X.R., Fang Y., Smith H.E. (2023). General practitioners’ perceptions of the roles of community pharmacists and their willingness to collaborate with pharmacists in primary care. J. Pharm. Policy Pract..

[15] Sanchez-Molina A.I., Benrimoj S.I., Ferri-Garcia R., Martinez-Martinez F., Gastelurrutia M.A., Garcia-Cardenas V. (2022). Development and validation of a tool to measure collaborative practice between community pharmacists and physicians from the perspective of community pharmacists: The professional collaborative practice tool. BMC Health Serv. Res..

[16] Griese-Mammen N., Hersberger K.E., Messerli M., Leikola S., Horvat N., van Mil J.W.F., Kos M. (2018). PCNE definition of medication review: Reaching agreement. Int. J. Clin. Pharm..

[17] Ogundipe A., Sim T.F., Emmerton L. (2023). The case to improve technologies for pharmacists’ prescribing. Int. J. Pharm. Pract..

[18] Bates I., Bader L.R., Galbraith K. (2020). A global survey on trends in advanced practice and specialisation in the pharmacy workforce. J. Pharm. Policy Pract..

[19] Anderson C., Bates I., Beck D., Brock T.P., Futter B., Mercer H., Rouse M., Whitmarsh S., Wuliji T., Yonemura A. (2009). The WHO UNESCO FIP Pharmacy Education Taskforce. Hum. Resour. Health.

[20] Dreischulte T., Fernandez-Llimos F. (2016). Current perceptions of the term Clinical Pharmacy and its relationship to Pharmaceutical Care: A survey of members of the European Society of Clinical Pharmacy. Int. J. Clin. Pharm..

[21] Batista J.P.B., Torre C., Sousa Lobo J.M., Sepodes B. (2022). A review of the continuous professional development system for pharmacists. Hum. Resour. Health.

[22] Koudmani D., Bader L.R., Bates I. (2024). Developing and validating development goals towards transforming a global framework for pharmacy practice. Res. Soc. Adm. Pharm..

[23] Udoh A., Bruno-Tomé A., Ernawati D.K., Galbraith K., Bates I. (2021). The development, validity and applicability to practice of pharmacy-related competency frameworks: A systematic review. Res. Soc. Adm. Pharm..

[24] Udoh A., Bruno-Tomé A., Ernawati D.K., Galbraith K., Bates I. (2021). The effectiveness and impact on performance of pharmacy-related competency development frameworks: A systematic review and meta-analysis. Res. Soc. Adm. Pharm..

[25] Meilianti S., Galbraith K., Bader L., Udoh A., Ernawati D., Bates I. (2023). The development and validation of a global advanced development framework for the pharmacy workforce: A four-stage multi-methods approach. Int. J. Clin. Pharm..

[26] Rushworth G.F., Innes C., Macdonald A., MacDonald C., McAuley L., McDavitt A., Stewart F., Bruce R. (2021). Development of innovative simulation teaching for advanced general practice clinical pharmacists. Int. J. Clin. Pharm..

[27] Zimonjić I., Marinković V., Mira J.J., Djokic B.B., Odalović M. (2025). Addressing the second victim phenomenon among community pharmacists and its impact on clinical pharmacy practice: A consensus study. Int. J. Clin. Pharm..

[28] Earle-Payne K., Forsyth P., Johnson C.F., Harrison H., Robertson S., Weidmann A.E. (2022). The standards of practice for delivery of polypharmacy and chronic disease medication reviews by general practice clinical pharmacists: A consensus study. Int. J. Clin. Pharm..

[29] The Pharmaceutical Chamber of Serbia Regulations. https://www.farmkom.rs/stranice/propisi/pravilnici.

[30] The Pharmaceutical Chamber of Serbia Register of Pharmacists. https://www.farmkom.rs/registar-farmaceuta.

[31] Chamber of Nurses and Healthcare Technicians of Serbia Register of Pharmacy Technicians. https://kmszts.org.rs/struktura-clanstva/.

[32] The Pharmaceutical Chamber of Serbia National Framework for the Assessment of Pharmacists’ Competencies. https://www.farmkom.rs/pdf/stranice/nacionalni-okviri-za-procenu-kompetencija-farmaceuta-final.pdf.

[33] The Pharmaceutical Chamber of Serbia Register of Pharmacists. https://www.farmkom.rs/pdf/projekti_pdf/fks-nacionalni-okvir-kompetencija.pdf.

[34] Stojkov S., Tadić I., Crnjanski T., Krajnović D. (2016). Assessment and self-assessment of the pharmacists’ competencies using the global competency framework (GbCF) in Serbia. Vojn. Pregl..

[35] Bartholomew L.K., Parcel G.S., Kok G. (1998). Intervention mapping: A process for developing theory- and evidence-based health education programs. Health Educ. Behav..

[36] Smidt A., Balandin S., Sigafoos J., Reed V.A. (2009). The Kirkpatrick model: A useful tool for evaluating training outcomes. J. Intellect. Dev. Disabil..

[37] Hoffmann T.C., Glasziou P.P., Boutron I., Milne R., Perera R., Moher D., Altman D.G., Barbour V., Macdonald H., Johnston M. (2014). Better reporting of interventions: Template for intervention description and replication (TIDieR) checklist and guide. BMJ.

[38] Blanco M., Prunuske J., DiCorcia M., Learman L.A., Mutcheson B., Huang G.C. (2022). The DoCTRINE Guidelines: Defined Criteria To Report Innovations in Education. Acad. Med..

[39] El-Awaisi A., Koummich S., Koraysh S., El Hajj M.S. (2022). Patient Safety Education in Entry to Practice Pharmacy Programs: A Systematic Review. J. Patient Saf..

[40] Friedman E.A., Bickford R., Bjork C., Campbell J., Cometto G., Finch A., Kane C., Wetter S., Gostin L. (2023). The global health and care worker compact: Evidence base and policy considerations. BMJ Glob. Health.

[41] McIsaac M., Buchan J., Abu-Agla A., Kawar R., Campbell J. (2024). Global Strategy on Human Resources for Health: Workforce 2030—A Five-Year Check-In. Hum. Resour. Health.

[42] Ballaram S., Perumal-Pillay V., Suleman F. (2024). A scoping review of continuing education models and statutory requirements for pharmacists globally. BMC Med. Educ..

[43] Bader L., Kusynová Z., Duggan C. (2019). FIP Perspectives: Realising global patient safety goals requires an integrated approach with pharmacy at the core. Res. Soc. Adm. Pharm..

[44] Bajis D., Al-Haqan A., Mhlaba S., Bruno A., Bader L., Bates I. (2023). An evidence-led review of the FIP global competency framework for early career pharmacists training and development. Res. Soc. Adm. Pharm..

[45] Aryee G.F.B., Amoadu M., Obeng P., Sarkwah H.N., Malcalm E., Abraham S.A., Baah J.A., Agyare D.F., Banafo N.E., Ogaji D. (2024). Effectiveness of eLearning programme for capacity building of healthcare professionals: A systematic review. Hum. Resour. Health.

[46] BinDhim N.F., Althumiri N.A., Albluwi R.A., Aljadhey H.S. (2024). Competencies, skills, and personal characteristics needed for pharmacy leaders: An in-depth interview. Saudi Pharm..

[47] Zimonjić I., Dražeta L., Milošević T. Development of business competencies among pharmacists through the “Galiverse” mobile application. Proceedings of the International Scientific Conference on Information Technology, Computer Science, and Data Science SINTEZA.

[48] Polit D.F., Beck C.T. (2006). The content validity index: Are you sure you know what’s being reported? Critique and recommendations. Res. Nurs. Health.

[49] Goodman L.A. (1961). Snowball Sampling. Ann. Math. Stat..

[50] Zimonjić I., Marinković V., Mira J.J., Knežević B., Djokic B.B., Bogavac-Stanojević N., Odalović M. (2025). The second victim experience and support tool: A cross-cultural adaptation, validation and psychometric evaluation of the Serbian version for pharmacy professionals (SR-SVEST-R). Int. J. Clin. Pharm..

[51] Tamiru H., Huluka S.A., Negash B., Hailu K., Mekonen Z.T. (2023). National Continuing Professional Development (CPD) training needs of pharmacists in Ethiopia. Hum. Resour. Health.

[52] Alnahar S.A., Darwish R.M., Al Qasas S.Z., Al Shabani M.M., Bates I. (2024). Identifying training needs of practising community pharmacists in Jordan—A self-assessment study. BMC Health Serv. Res..

[53] Safwan J., Akel M., Sacre H., Haddad C., Sakr F., Hajj A., Zeenny R.M., Iskandar K., Salameh P. (2023). Academic pharmacist competencies in ordinary and emergency situations: Content validation and pilot description in Lebanese academia. BMC Med. Educ..

[54] Zeineddine L., Sacre H., Haddad C., Zeenny M.R., Akel M., Hajj A., Salameh P. (2023). The association of management and leadership competencies with work satisfaction among pharmacists in Lebanon. J. Pharm. Policy Pract..

[55] European Platform for Vocational Excellence in Health Care Innovation and Development of the Healthcare Sector. https://euveca.eu/.

[56] Blueprint Alliance for a Future Health Workforce Strategy on Digital and Green Skills Shaping the Health and Care Workforce of Tomorrow. https://bewell-project.eu/.

[57] Zimonjić I., Marinković V., Jocić D., Dražeta L., Odalović M. (2025). Perceived importance of tailored education to prevent second victim phenomenon in clinical pharmacy practice: A focus group study with community pharmacists. Int. J. Clin. Pharm..

