# Supplementary material for: Developing Soft Skills for Sustainable Community Pharmacy Practice Through a Competency-Based Modular Programme
Source: Pharmacy (Basel). 2025 Aug 20;13(4):110. doi: 10.3390/pharmacy13040110 (PMC12389078; doi:10.3390/pharmacy13040110)
Supplement: Supplementary file 1 [file pharmacy-13-00110-s001.zip › pharmacy-3776791-supplementary.pdf]

Supplementary Materials S1. Galenika Academy satisfaction questionnaire

1. Are you aware that you have attended ≥ 80% of the education within the Galenika Academy?

   a) Yes

   b) No

2. How much are you satisfied with the content of Galenika Academy?

   1 - Very dissatisfied
   2 - Dissatisfied
   3 - Neutral
   4 - Satisfied
   5 - Very satisfied

3. How much are you satisfied with the quality of education and the choice of lecturers?

   1 - Very dissatisfied
   2 - Dissatisfied
   3 - Neutral
   4 - Satisfied
   5 - Very satisfied

4. Are you aware that Galenika Academy covers organisational, managerial, personal, and professional competencies from the FIP Global Competency Framework?

   a) Yes

   b) No

5. Have you completed accredited tests on business skills?

   a) Yes

   b) No

6. How much are you satisfied with the content of the materials provided for test preparation?

   1 - Very dissatisfied
   2 - Dissatisfied
   3 - Neutral
   4 - Satisfied
   5 - Very satisfied

7. Have you heard of the Galiverse mobile application?

   a) Yes

b) No

8. Do you use the Galiverse mobile application?

   a) Yes

   b) No

9. How much are you satisfied with the mobile application?

   1 - Very dissatisfied
   2 - Dissatisfied
   3 - Neutral
   4 - Satisfied
   5 - Very satisfied

10. Do you perceive that the programme(s) you attended improved your competence?

    a) Yes

    b) No

11. How much are you satisfied with how you have improved your resilience through the educational programme? How satisfied are you with the way you have incorporated resilience-building strategies into your professional practice after the educational programme?

    1 - Very dissatisfied
    2 - Dissatisfied
    3 - Neutral
    4 - Satisfied
    5 - Very satisfied

12. How much are you satisfied with how education impacted the success and sustainability of your organisation? How satisfied are you with the way your behaviour has contributed to the success and sustainability of your organisation after the educational programme?

    1 - Very dissatisfied
    2 - Dissatisfied
    3 - Neutral
    4 - Satisfied
    5 - Very satisfied

13. Would you recommend an educational program to colleagues who wish to develop professionally?

    a) Yes

    b) No

14. Do you have any comments, suggestions, or feedback regarding the development programme you attended?

____

Demografic data

1. Sex:

a) Male
b) Female

2. Age (enter the number of years): ____________________

3. How many years of work experience do you have? (enter the number of years): ____________________

4. What is your highest level of education?

a) Secondary School
b) Collage Bachelor's Degree
c) University – Master's Degree
d) Specialised Academic Studies
e) Health Specialisation
f) Master's or Doctorate

5. What is your current job position?

a) Pharmacist
b) Pharmacy technician

6. In which type of institution do you work?

a) Private Pharmacy
b) State Pharmacy

7. The healthcare institution where you are employed is located in:

a) Rural area
b) Smaller town (up to 100,000 inhabitants)
c) Medium-sized city (100-200,000 inhabitants)
d) Large city (over 200,000 inhabitants)

Supplementary Materials S2. Summary of webinars and participants in live sessions online.

Webinar topics	Competence cluster [1]	Competencies	Number of participants
1. "Small school of resilience for healthcare professionals – burnout"	1. Pharmaceutical Public Health, 4. Professional/Personal	1.1 Emergency response, 4.5 Leadership and self-regulation, 4.7 Professional and ethical practice	787
2. "Small school of resilience for healthcare professionals – second victims"	1. Pharmaceutical Public Health, 4. Professional/Personal	1.1 Emergency response, 4.5 Leadership and self-regulation	692
3. "Teamwork in healthcare"	1. Pharmaceutical Public Health, 3. Organisation and Management	3.2 Human resources management	563
4. "Small school of resilience for healthcare professionals – resilience"	1. Pharmaceutical Public Health, 4. Professional/Personal	1.1 Emergency response, 4.5 Leadership and self-regulation	452
5. "Communication in healthcare"	1. Pharmaceutical Public Health, 4. Professional/Personal	4.1 Communication skills	438
6. "Quality in pharmaceutical care"	4. Professional/Personal	4.2 Continuing Professional Development (CPD), 4.8 Quality assurance and research in the workplace	433
7. "Why is time management important for pharmacists in pharmacies?"	3. Organisation and Management, 4. Professional/Personal	3.2 Human resources management, 3.6 Workplace management, 4.5	752

		Leadership and self-regulation	
8. "Risk management in pharmaceutical care"	1. Pharmaceutical Public Health, 3. Organisation and Management, 4. Professional/Personal	1.1 Emergency response, 3.3 Improvement of service, 3.4 Procurement, 3.5 Supply chain management, 4.5 Leadership and self-regulation, 4.6 Legal and regulatory practice	734
9. "Why are digital communication and leadership important for pharmacists?"	4. Professional/Personal	4.1 Digital literacy, 4.5 Leadership and self-regulation	650
10. "Why is negotiation important for pharmacists?"	3. Organisation and Management	3.1 Budget and reimbursement, 3.4 Procurement, 3.5 Supply chain management	570
11. "Why team development is important for pharmacists"	3. Organisation and Management	3.2 Human resources management	558
12. "How to motivate employees in pharmacies?"	3. Organisation and Management, 4. Professional/Personal	3.2 Human resources management, 3.6 Workplace management, 4.5 Leadership and self-regulation	514
13. "Why a business plan is important for a new service in a pharmacy"	3. Organisation and Management	3.1. Budget and reimbursement, 3.2 Human resources management, 3.3	446

		Improvement of service	
14. "Why conflict management is important for pharmacists"	4. Professional/Personal	4.4 Interprofessional collaboration	410
15. "Why is communication important for pharmacists in pharmacies?"	4. Professional/Personal	4.1 Communication skills, 4.4 Interprofessional collaboration	375
16. "Why is business continuity management important in pharmacies?"	3. Organisation and Management, 4. Professional/Personal	3.1. Budget and reimbursement, 3.6 Workplace management, 4.8 Quality assurance and research in the workplace	286
17. "Pharmacy as a business system and the development of personal skills"	3. Organisation and Management	3.1 Budget and reimbursement, 3.2 Human resources management, 3.6 Workplace management	269
18. "Why is research in pharmaceutical practice important for pharmacists?"	3. Organisation and Management, 4. Professional/Personal	3.3 Improvement of service, 4.2 Continuing Professional Development (CPD), 4.8 Quality assurance and research in the workplace, 4.3 Digital literacy, 4.4 Interprofessional collaboration	250
19. "Tools for problem solving and business	1. Pharmaceutical Public Health, 3. Organisation and	1.1 Emergency response, 3.3 Improvement of	239

decision-making in pharmacies"	Management, 4. Professional/Personal	services, 3.6 Workplace management, 4.7 Professional and ethical practice	
20. "Why is performance management important for pharmacy professionals?"	3. Organisation and Management, 4. Professional/Personal	3.2 Human resources management, 3.6 Workplace management, 4.2 Continuing Professional Development (CPD)	189
21. "Why is employee development important for the business of pharmacies?"	3. Organisation and management, 4. Professional/Personal	3.2. Human resources management, 4.2 Continuing Professional Development (CPD)	172
22. "Pharmacy marketing mix and brand management"	4. Professional/Personal	4.6 Legal and regulatory practice, 4.7 Professional and ethical practice	171
23. "Regulations in advertising in pharmacies"	4. Professional/Personal	4.6 Legal and regulatory practice, 4.7 Professional and ethical practice	167
24. "Project management in pharmaceutical practice"	3. Organisation and Management, 4. Professional/Personal	3.1. Budget and reimbursement, 3.6 Workplace management, 4.8 Quality assurance and research in the workplace	158
25. "Why are professionalism and ethics important for pharmacy professionals?"	4. Professional/Personal	4.6 Legal and regulatory practice, 4.7 Professional and ethical practice	152

Total		10,427

[1] Individual competencies are categorised into clusters, and expected behaviours are presented in alignment with the International Pharmaceutical Federation (FIP) Global Competency Framework – Early Career Training Version 2. Test passed ≥60% – participants who scored ≥60% on the final knowledge assessment.

Supplementary Materials S3. Summary of accredited tests: topics, participant numbers, and pass rates.

Education - accredited test	Accreditation number [2]	Expected learning time	Number of questions	Number of points for licence renewal	Number of participants	Number of successfully completed (>60% of questions with correct answers)	Success rate (% of enrolled)
1. "Pharmacy as a business system and development of personal abilities"	B-74/23, Re-accreditation B-41/24	2 hours for learning, 30 minutes for test completion	20	2	725	605	83.4%
2. "Time management in pharmaceutical organisations - pharmacies"	B-88/21, Re-accreditations: B-122/22, B-161/23	4 hours for learning, 1 hour for test completion	40	4	762	654	85.8%
3. "Creation and management of teams in pharmaceutical organisations - pharmacies"	B-89/21, Re-accreditations: B-118/22, B-157/23	4 hours for learning, 1 hour for test completion	40	4	479	292	61.0%
4. "Management of work performance in the pharmacy"	B-75/23, Re-accreditation B-44/24	2 hours for learning, 30 minutes for test completion	20	2	572	498	87.1%

5.	"Teamwork of health professionals as a prerequisite for patient safety"	B-20/23, Re-accreditation B-6/24	4 hours for learning, 1 hour for test completion	40	4	928	722	77.8%
6.	"Decision-making and problem-solving in pharmaceutical practice"	B-73/23, Re-accreditation B-46/24	3 hours for learning, 45 minutes for test completion	30	3	521	436	83.7%
7.	"Business negotiation in pharmaceutical practice"	B-79/21, Re-accreditations: B-119/22, B-159/23	4 hours for learning, 1 hour for test completion	40	4	291	216	89.7%
8.	"Risk management in pharmaceutical practice"	B-22/23, Re-accreditation B-11/24	2 hours for learning, 20 minutes for test completion	20	2	555	470	84.7%
9.	"Communication of health professionals as a prerequisite for patient safety"	B-21/23, Re-accreditation B-7/24	3 hours for learning, 45 minutes for test completion	30	3	813	694	85.4%
10.	"Research in pharmaceutical practice"	B-77/23, Re-accreditation B-45/24	2 hours for learning, 20 minutes for test completion	20	2	371	297	80.1%
11.	"Healthcare professionals as second victims of negative outcomes in health care"	B-31/23, Re-accreditation B-10/24	2 hours for learning, 30 minutes for test completion	20	2	682	544	79.8%

12.	"Development of employees in the pharmacy"	B-76/23, Re-accreditation B-40/24	2 hours for learning, 30 minutes for test completion	20	2	361	292	80.9%
13.	"Resilience of health professionals as a prerequisite for patient safety"	B-29/23, Re-accreditation B-9/24	2 hours for learning, 30 minutes for test completion	20	2	506	482	95.3%
14.	"Burnout syndrome in health practice"	B-30/23, Re-accreditation B-8/24	2 hours for learning, 30 minutes for test completion	20	2	546	516	94.5%
15.	"Standards for establishing quality and safety in pharmaceutical practice"	B-78/23, Re-accreditation B-39/24	2 hours for learning, 30 min for completion	20	2	140	126	90.0%
Total (number of participants, % of success rate for all tests)						8,252	6,844	82.9%

[2] Health Council of the Republic of Serbia approval and Pharmaceutical Chamber of Serbia accreditation for working licence renewal. Professional licences are renewed every seven years, requiring the accumulation of 140 continuing education points over the period, with a minimum of 10 points earned per working year.

Supplementary Materials S4. Galenika Academy satisfaction questionnaire results (participants attended ≥ 80% of the education).

Variable		How much are you satisfied with the content of Galenika Academy?					P* Value	How much are you satisfied with the quality of education and the choice of lecturers?					p Value	How much are you satisfied with the content of the materials provided for test preparation?					p Value	How much are you satisfied with the mobile application?					p Value	How much are you satisfied with how education improved your business sustainability?					P Value
		Number (%) of participants, n=222																													
		1 - Very dissatisfied	2 - Dissatisfied	3 - Neutral	4 - Satisfied	5 - Very satisfied		1 - Very dissatisfied	2 - Dissatisfied	3 - Neutral	4 - Satisfied	5 - Very satisfied		1 - Very dissatisfied	2 - Dissatisfied	3 - Neutral	4 - Satisfied	5 - Very satisfied		1 - Very dissatisfied	2 - Dissatisfied	3 - Neutral	4 - Satisfied	5 - Very satisfied		1 - Very dissatisfied	2 - Dissatisfied	3 - Neutral	4 - Satisfied	5 - Very satisfied	
Are you aware that you have attended ≥ 80% educations within the Galenika Academy?	Yes	1 (0.5%)	0 (0.0%)	5 (2.6%)	25 (12.9%)	163 (84%)	**<0.001**	1 (0.5%)	0 (0.0%)	5 (2.6%)	23 (11.9%)	165 (85.1%)	**<0.001**	3 (1.5%)	2 (1.0%)	7 (3.6%)	31 (16.0%)	151 (77.8%)	0.096	12 (6.2%)	7 (3.6%)	24 (12.4%)	32 (16.5%)	119 (61.3%)	0.603	1 (0.5%)	1 (0.5%)	23 (11.9%)	35 (18.0%)	134 (69.1%)	0.684
	No	0 (0.0%)	1 (3.6%)	3 (10.7%)	8 (28.6%)	16 (57.1%)		0 (0%)	1 (3.6%)	6 (2.4%)	4 (14.3%)	17 (60.7%)		2 (7.1%)	1 (3.6%)	2 (7.1%)	7 (25%)	16 (27.1%)		3 (10.7%)	1 (3.6%)	4 (14.3%)	7 (25.0%)	13 (46.4%)		0 (0.0%)	0 (0.0%)	6 (21.4%)	4 (14.3%)	18 (64.3%)	
Have you heard of the Galiverse application?	Yes	0 (0.0%)	1 (0.6%)	5 (2.9%)	26 (15.2%)	139 (81.3%)	0.319	0 (0.0%)	1 (0.6%)	5 (2.9%)	20 (11.7%)	145 (84.8%)	**0.030**	1 (0.6%)	3 (1.8%)	8 (4.7%)	27 (15.8)	132 (77.2%)	**0.018**	5 (2.9%)	5 (2.9%)	13 (7.6%)	33 (19.3%)	115 (67.3%)	**<0.001**	0 (0.0%)	1 (0.6%)	18 (10.5%)	32 (18.7%)	120 (70.2)	0.085
	No	1 (2.0%)	0 (0.0%)	3 (5.9%)	7 (13.7%)	40 (78.4)		1 (2.0%)	0 (0.0.%)	6 (11.8%)	7 (13.7%)	37 (72.5%)		4 (7.8%)	0 (0.0%)	1 (2.0%)	11 (21.6%)	35 (68.6%)		10 (19.3%)	3 (5.9%)	15 (29.4%)	6 (11.8%)	17 (33.3%)		1 (2.0%)	0 (0.0%)	11 (21.6%)	7 (13.6%)	32 (32.7%)	
Do you use the Galiverse application?	Yes	0 (0.0%)	0 (0.0%)	4 (3.2%)	16 (12.8%)	105 (84.0%0	0.416	0 (0.0%)	0 (0.0%)	4 (3.2%)	14 (11.2%)	107 (85.6%)	0.284	0 (0.0%)	2 (1.6%)	6 (4.8%)	19 (15.2%)	98 (78.4%)	0.093	0 (0.0%)	1 (0.8%)	3 (2.4%)	29 (23.2%)	92 (73.6%)	**<0.001**	0 (0.0%)	1 (0.8%)	11 (8.8%)	24 (19.2%)	89 (71.2%)	0.147
	No	1 (0.5%)	1 (0.5%)	4 (4.1%)	17 (17.5%)	74 (76.3%)		1 (1.0%)	1 (1.0%)	7 (7.2%)	13 (13.4%)	75 (77.3%)		5 (5.2%)	1 (1.0%)	3 (3.1%)	19 (19.6%)	69 (71.1%)		15 (15.5%)	7 (7.2%)	25 (25.8%)	10 (10.3%)	40 (40.1%)		1 (1.0%)	0 (0.0%)	18 (18.6%)	15 (15.5%)	63 (64.9%)	
Do you perceive that the programme(s) you attended improved your competence?	Yes	1 (0.5%)	1 (0.5%)	7 (3.3%)	30 (14.1%)	174 (81.7%)	0.350	1 (0.5%)	1 (0.5%)	9 (4.2%)	24 (11.3%)	178 (83.6%)	**0.029**	4 (1.9%)	3 (1.4%)	7 (3.3%)	35 (16.4%)	164 (77.0%)	**0.005**	11 (5.2%)	8 (3.8%)	26 (12.2%)	38 (17.8%)	130 (61.0%)	**<0.001**	1 (0.5%)	1 (0.05%)	25 (11.7%)	37 (17.4%)	149 (70.0%)	0.062
	No	0 (0.0%)	0 (0.0%)	1 (11.1%)	3 (33.3%)	5 (55.6%)		0 (0.0%)	0 (0.0%)	2 (22.2%)	3 (33.3%)	4 (44.4%)		1 (11.1%)	0 (0.0%)	2 (22.2%)	3 (33.3%)	3 (33.3%)		4 (44.4%)	0 (0.0%)	2 (22.2%)	1 (11.1%)	2 (22.2%)		0 (0.0%)	0 (0.0%)	4 (44.4%)	2 (22.2%)	3 (33.3%)	
Would you recommend an educational program to colleagues who wish to develop professionally?	Yes	1 (0.5%)	1 (0.5%)	6 (2.8%)	33 (15.2%)	176 (81.1%)	**<0.001**	1 (0.5%)	1 (0.5%)	9 (4.1%)	27 (12.4%)	179 (82.5%)	**0.009**	4 (1.8%)	2 (0.9%)	9 (4.1%)	38 (17.5%)	164 (75.6%)	**<0.001**	15 (6.9%)	8 (3.7%)	26 (12.0%)	39 (18.0%)	129 (29.4%)	0.346	1 (0.5%)	1 (0.5%)	27 (12.4%)	38 (17.5%)	150 (69.1%)	0.476
	No	0 (0.0%)	0 (0.0%)	2 (40%)	0 (0.0%)	3 (60.0%)		0 (0.0%)	0 (0.0%)	2 (40.0%)	0 (0.0%)	3 (60.0%)		1 (20.0%)	1 (20.0%)	0 (0.0%)	0 (0.0%)	3 (60.0%)		0 (0.0%)	0 (0.0%)	2 (40.0%)	0 (0.0%)	3 (60.0%)		0 (0.0%)	0 (0.0%)	2 (40.0%)	1 (20.0%)	2 (40.0%)	
How much are you	1 - Very dissatisfied	0 (0.0%)	0 (0.0%)	0 (0.0%)	0 (0.0%)	2 (100.0 %)	**<0.001**	0 (0.0%)	0 (0.0%)	0 (0.0%)	0 (0.0%)	2 (100.0 %)	**<0.001**	0 (0.0%)	0 (0.0%)	1 (50.0 %)	0 (0.0%)	1 (50.0 %)	**<0.001**	1 (50.0%)	0 (0.0%)	0 (0.0%)	0 (0.0%)	1 50.0%)	**<0.001**	1 (50.0%)	0 (0.0%)	0 (0.0%)	1 (50.0 %)	0 (0.0%)	**<0.001**

satisfied with how you have improved your resilience through the educational program?	2 - Dissatisfied	0 (0.0%)	1 (20.0%)	1 (20.0%)	2 (40.0%)	1 (20.0%)	0 (0.0%)	1 (20.0%)	1 (20.0%)	2 (40.0%)	1 (20.0%)	0 (0.0%)	2 (40.0%)	0 (0.0%)	2 (40.0%)	1 (20.0%)	0 (0.0%)	1 20.0%)	0 (0.0%)	2 (40.0%)	2 (40.0%)	0 (0.0%)	0 (0.0%)	1 (20.0%)	2 (40.0%)	2 (40.0%)
	3 - Neutral	0 (0.0%)	0 (0.0%)	5 12.8%)	15 (28.5%0	19 (48.7%)	0 (0.0%)	0 (0.0%)	7 (17.9%)	13 (33.3%)	19 (48.7%)	3 (7.7%)	1 (2.6%)	5 (12.8%)	18 (46.2%)	12 (30.8%)	6 (15.4%)	2 (5.1%)	12 (30.8%)	12 (30.8%)	7 (17.9%)	0 (0.0%)	1 (2.6%)	23 (29.0%)	11 (28.2%)	4 (10.3%)
	4 - Satisfied	0 (0.0%)	0 (0.0%)	1 (2.1%)	8 (17.0%)	38 (80.9%)	0 (0.0%)	0 (0.0%)	3 (6.4%)	7 (14.9%)	37 (78.7%)	0 (0.0%)	0 (0.0%)	1 (2.1%)	10 (21.3%)	36 (76.6%)	3 (6.4%)	2 (4.3%)	10 (21.3%)	13 (27.7%)	19 (40.4%)	0 (0.0%)	0 (0.0%)	5 (10.6%)	21 (44.7%)	21 (44.7%)
	5 - Very satisfied	1 (0.8%)	0 (0.0%)	1 (0.8%)	8 (6.2%)	119 (92.2%)	1 (0.5%)	1 (0.5%)	11 (5.0%)	27 (12.2%)	182 (82.0%)	2 (1.6%)	0 (0.0%)	2 (1.6%)	8 (6.2%)	117 (90.7%)	5 (3.9%)	3 (2.3%)	6 (4.7%)	12 (9.3%)	103 (79.8%)	0 (0.0%)	0 (0.0%)	0 (0.0%)	4 (3.1%)	125 (96.9%)

* Bold typeface indicates significant p values (p<0.05).
